# Hanging drop sample preparation improves sensitivity of spatial proteomics†

**DOI:** 10.1039/d2lc00384h

**Published:** 2022-07-12

**Authors:** Yumi Kwon, Paul D. Piehowski, Rui Zhao, Ryan L. Sontag, Ronald J. Moore, Kristin E. Burnum-Johnson, Richard D. Smith, Wei-Jun Qian, Ryan T. Kelly, Ying Zhu

**Affiliations:** a Environmental Molecular Sciences Laboratory, Pacific Northwest National Laboratory 902 Battelle Boulevard Richland WA 99354 USA ying.zhu@pnnl.gov; b Biological Sciences Division, Pacific Northwest National Laboratory Richland WA 99354 USA; c Department of Chemistry and Biochemistry, Brigham Young University C100 BNSN Provo UT 84602 USA

## Abstract

Spatial proteomics holds great promise for revealing tissue heterogeneity in both physiological and pathological conditions. However, one significant limitation of most spatial proteomics workflows is the requirement of large sample amounts that blurs cell-type-specific or microstructure-specific information. In this study, we developed an improved sample preparation approach for spatial proteomics and integrated it with our previously-established laser capture microdissection (LCM) and microfluidics sample processing platform. Specifically, we developed a hanging drop (HD) method to improve the sample recovery by positioning a nanowell chip upside-down during protein extraction and tryptic digestion steps. Compared with the commonly-used sitting-drop method, the HD method keeps the tissue pixel away from the container surface, and thus improves the accessibility of the extraction/digestion buffer to the tissue sample. The HD method can increase the MS signal by 7 fold, leading to a 66% increase in the number of identified proteins. An average of 721, 1489, and 2521 proteins can be quantitatively profiled from laser-dissected 10 μm-thick mouse liver tissue pixels with areas of 0.0025, 0.01, and 0.04 mm^2^, respectively. The improved system was further validated in the study of cell-type-specific proteomes of mouse uterine tissues.

## Abstract 2

An improved spatial proteomics platform to quantify >1500 proteins at a high spatial resolution based on a hanging-drop arrangement during protein extraction and digestion.

## Graphical abstract

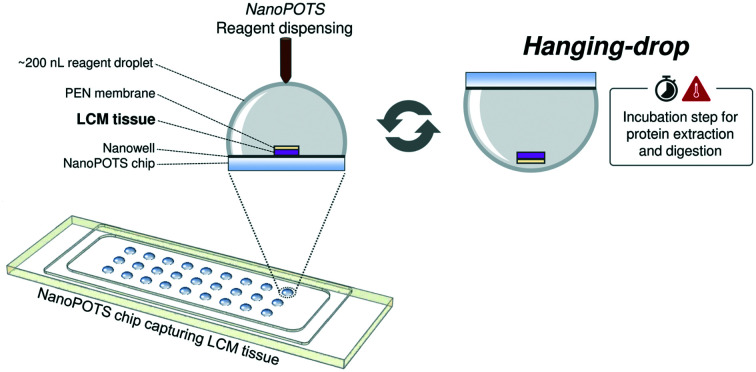

## Introduction

Human organs and tissues are highly complex machineries, containing many different cell types organized in spatially defined patterns. The organization of cell populations, as well as cell–cell and cell–matrix interactions, have critical impacts in physiological and pathological tissue environments.^1–3^ Many technologies have been developed to characterize the spatial distribution of biomolecules in human tissues. Among them, immunohistochemistry and immunofluorescence are the most widely used to visualize the two or three-dimensional patterns of protein abundances across various cell types. The integration of immunostaining with mass spectrometry (*e.g.*, mass cytometry imaging^4^ and multiplexed ion beam imaging^5^) has increased the number of proteins (∼40) that can be visualized simultaneously. However, this only represents <1% of proteins typically expressed in human cells and thus significantly limits our ability to study the tissue microenvironment in a systematic way.

Mass spectrometry (MS)-based proteomics has been demonstrated to characterize >10 000 proteins in human tissues without the requirement of antibodies.^6^ To enable spatial proteomics analysis of tissue sections, several microsampling approaches have been developed,^7^ including manual dissection,^8^ solvent extraction,^9,10^*in situ* digestion with microdroplet^11–14^ or hydrogel,^15–18^ and laser capture microdissection (LCM).^19–23^ The coupling of these microsampling approaches with nanoscale liquid chromatography-mass spectrometry (LC-MS) has enabled the proteome profiling of ∼500 proteins from tissue regions as small as 0.1 mm^2^ and 10 μm thickness in diameters.^23^ Deep proteome coverage required significantly large tissue areas of >1.5 mm in diameter.^24^ Thus, there is a great need to improve the overall sensitivity of spatial proteomics to enable deeper proteome coverage at high resolution.

To address these challenges, we recently combined LCM-based microsampling with a microfluidic sample preparation method, nanoPOTS (nanodroplet processing in one pot for trace samples).^25^ NanoPOTS significantly improves the overall sensitivity of proteomic analyses by performing protein extraction and digestion in <200 nL droplets.^25^ The use of nanoliter droplets not only significantly reduces surface-adsorption-related sample losses, but also improves tryptic digestion kinetics. To enable the automated integration of nanoPOTS with LCM, we developed a DMSO-mediated tissue collection method to reliably capture microdissected tissue pixels in droplet arrays.^26^ We demonstrated that the LCM-nanoPOTS-based spatial proteomics not only provides cell-specific proteome profiles, but also facilitates the generation of unbiased proteome-scale maps across the tissue section.^27^ The platform has been widely used to study the cell-type-specific proteomes in rat and human brains,^26,28^ mouse uterus,^27^ tomato fruit,^29^ and poplar leaf and root.^30^

Herein, we describe an improved LCM-nanoPOTS spatial proteomics workflow. Specifically, we developed a hanging-drop (HD)-based sample preparation approach by simply positioning the nanoPOTS chip upside-down during protein extraction and tryptic digestion steps. The HD approach shares similarities with three-dimensional (3D) cell culture^31–33^ and HD protein crystallization methods.^34–37^ In both methods, the HD arrangement is employed to avoid the direct contact of sample with container surface, minimize the complicated sample/solid interactions, and thus improve the spheroid formation and crystal growth, respectively. In this study, the HD arrangement is used to keep tissue pixel away from nanowell surfaces to improve the accessibility of the extraction/digestion buffer to tissue sample, and thus to enhance the sample recovery of spatial proteomics. We observed a significant improvement in proteome coverage and sensitivity for LCM-isolated tissue pixels using HD sample preparation compared with the conventional sitting-drop (SD) approach. The improved system was further evaluated in a study of cell-type-specific proteomes in mouse uterine section.

## Experimental

### Chemicals and materials

Dithiothreitol (DTT) and iodoacetamide (IAA) were obtained from Thermo Fisher Scientific (Rockford, IL, USA). Ammonium bicarbonate (ABC), *n*-dodecyl-β-d-maltoside (DDM), Mayer's hematoxylin, eosin Y (alcoholic solution), Scott's Tap Water Substitute and dimethyl sulfoxide (DMSO, HPLC grade) were purchased from Sigma-Aldrich (St. Louis, MO, USA). RapiGest SF surfactant was from Waters (Waters, Milford, MA, USA). ProteaseMAX Surfactant, Trypsin (MS grade), and Lys-C (MS grade) were from Promega (Madison, WI, USA).

### Mouse tissue preparation

All procedures involving animals in this study were in accordance with protocols established under NIH and institutional guidelines for the use of laboratory animals and were reviewed by the Institutional Animal Care and Use Committee of Battelle, Pacific Northwest Division. For mouse liver tissue, C57BL/6J mice were obtained from Jackson Labs. Mouse liver was prepared for LCM as previously described.^27^ Briefly, after harvesting liver from mouse, tissues were washed with phosphate buffer saline, flash-frozen in liquid N_2_ and stored at −80 °C until use. A cryostat (NX-70, Thermo Fisher Scientific) was used to section tissues at a thickness of 10 μm, which were mounted on polyethylene naphthalate (PEN) membrane slides (Carl Zeiss Microscopy, Germany), and stored at −80 °C until use. Mouse uterus from *Wnt5a*^d/d^ mice was provided by Prof. Sudhansu K. Dey in Cincinnati Children's Hospital Medical Center.^27,38^ Uterine tissues were sectioned at a thickness of 12 μm, mounted on PEN membrane slides, and stored at −80 °C until use. To fix proteins, after removal from −80 °C freezer, tissue slides were immediately immersed into pre-cooled tissue fixative solution (70% ethanol) for 15 s. The tissue slides were then rehydrated for 30 s in deionized water and stained with Mayer's hematoxylin solution (Sigma-Aldrich, St. Louis, USA). Next, the stained sections were dehydrated with 70%, 95%, and 100% ethanol for 30 s, 1 min, and 1 min, respectively. The dehydrated sections were dried in a fume hood for 10 min, which can be used directly or stored at −80 °C until use.

### Nanowell chip fabrication

Nanowell chips were fabricated on glass slides as described previously.^25^ Briefly, an array of 3 × 9 nanowells with a diameter 1.2 mm and a center-to-center spacing of 4.5 mm were fabricated on glass slides with pre-coated chromium and photoresist (25 mm × 75 mm, Telic company, Valencia, USA), based on standard photolithography and wet etching. After wet etching, the remaining glass surfaces were treated with 2% (v/v) heptadecafluoro-1,1,2,2-tetrahydrodecyldimethylchlorosilane in 2,2,4-trimethylpentane. After removing the remaining chromium layer, an array of spots that maintained hydrophilicity served as nanowells for tissue collection and proteomic sample processing.

### Laser capture microdissection of tissue sections

Before LCM, a 200 nL of DMSO droplet was preloaded into each nanowell. LCM was performed on a PALM MicroBeam system (Carl Zeiss, Munich, Germany). Pixelation of the tissue section was achieved by first drawing a square on the tissue using PalmRobo software, followed by tissue cutting and catapulting. Both liver and uterine tissues were cut at an energy level of 42 and catapulted into the DMSO droplet on nanowells using the “CenterRoboLPC” function with an energy level of delta 15 and a focus level of delta 5. The collected samples on nanowells can be processed directly or stored at −20 °C for weeks.

### Proteomic sample processing

**Fig. 1:**
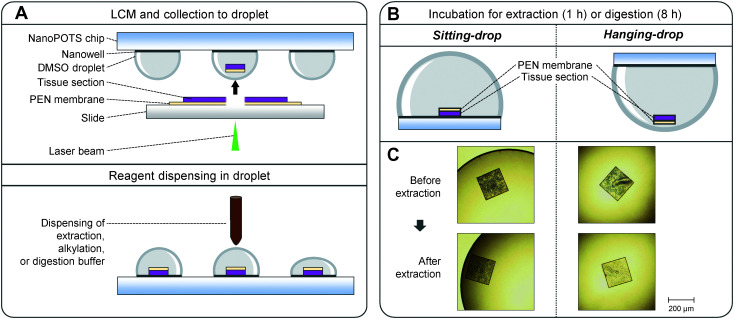
LCM-nanoPOTS-based spatial proteomics with sitting drop (SD) and hanging drop (HD) approaches. (A) Illustration of the DMSO-mediated tissue pixel capture and nanoPOTS protocol for proteomic sample preparation. (B) Schematic diagram of side views showing the location of tissue pixels during the incubation steps based on SD and HD approaches. (C) Representative microscopic images of LCM-dissected tissue pixels before and after protein extraction step. The LCM tissues processed with HD approach exhibit less tissue staining color than that with SD approach, indicating improved protein extraction efficiency.

Before processing, the DMSO droplets were evaporated to dryness for ∼30 min using a vacuum desiccator. A home-built nanoliter-scale robotic liquid-handling platform was employed to dispense reagents into nanowells.^25,39^ Briefly, 100 nL of cell lysis buffer (0.1% (w/v) DDM, 5 mM DTT, and 1× PBS) was applied to each nanowell. To evaluate different extraction buffers, 0.1% or 0.5% of DDM, 0.1% or 0.5% of ProteaseMAX, or 0.1% or 0.5% of RapiGest buffers with 1× PBS supplemented with 5 mM DTT were tested. The chip was incubated at 70 °C for 1 h. During the extraction and the following alkylation and digestion steps, the chip was placed in an upside-down direction to implement the HD method (Fig. 1). Next, 50 nL of 30 mM iodoacetamide in 50 mM ammonium bicarbonate (ABC) buffer (pH 8.0) was added to each well and incubated in the dark for 30 min. Protein digestion was performed at 37 °C by dispensing 50 nL of 0.01 ng nL^−1^ Lys-C (MS grade, Promega, Madison, USA) and 0.04 ng nL^−1^ trypsin (Promega) in ABC buffer and incubating for 8 h. Finally, the digestion was quenched by adding 50 nL of 5% formic acid (FA) and incubated for 15 min. Finally, the droplets were transferred into 96-well plates (PCR Plates; Eppendorf, Hauppauge, USA). Prior to droplet transfer, the PCR plate was prefilled with 25 μL aqueous buffer containing 0.1% FA and 0.02% DDM. The 96-well plate was sealed with a PCR sealing membrane and stored in −20 °C.

### Sample analysis with LC-MS/MS

A home-built LC system was used to perform sample injection and LC separation.^40^ The system was built on a PAL autosampler (CTC Analytics AG, Switzerland). Two six-port valves (Valco Instruments Co. Inc., Houston, TX) were used to perform the sample injection and capillary-based solid-phase extraction (SPE). The sample in 96-well plate was loaded into a 25 μL sample loop and then purified with a capillary SPE (150 μm i.d., 4 cm length, slurry-packed with Jupiter C18 packing material, 300 Å pore size, 5 μm particle sizes; Phenomenex, Torrance, CA, USA). For separation, LC column was prepared on a 60 cm, 50 μm-i.d. fused-silica capillary (3 μm diameter particle, 300 Å pore size C18 particles, Phenomenex, Torrance, CA) with an integrated emitter (Self-Pack PicoFrit column, New Objective, Woburn, USA).

A nanoUPLCpump (Dionex UltiMate NCP-3200RS, Thermo Scientific, Waltham, MI) was used to deliver mobile phase (buffer A: 0.1% formic acid in water; buffer B: 0.1% formic acid in acetonitrile) at 150 nL min^−1^. A linear 100 min gradient started from 8% buffer B to 22%, followed by a 15 min linear increase to 35% buffer B. The column was washed with 90% buffer B for 5 min and re-equilibrated with 2% buffer B for 20 min prior to the subsequent analysis. A Q Exactive plus hybrid quadrupole-Orbitrap mass spectrometer (Thermo Scientific) was used to collect the data. Electrospray voltage at the ionization source was set to 2.2 kV, and the temperature for ion transfer tube was set to 250 °C. The S-lens RF level was set at 70. Data-dependent acquisition was employed to automatically switch between full-scan MS and top-12 MS/MS scans. Full scans were obtained at a resolution of 35 000, an AGC target of 3E6, a maximum injection time of 100 ms, and a mass range of 300–1800 *m*/*z*. Precursor ions with charges from +2 to +7 isolated with an isolation window of 2, an AGC target of 1E5, a maximum injection time 100 ms were sequentially fragmented by high energy dissociation (HCD) with a collision energy of 30%, and scanned in orbitrap at a resolution of 17 500.

### Data analysis

All raw files were processed using FragPipe (v17.1) with MSFragger^41,42^ (v.3.4) search engine against a Uniprot mouse (*Mus musculus*) database (11/22/2021 release) containing 16 969 mouse sequences, common contaminant sequences, and decoy protein sequences. MS/MS spectra were searched with the following search parameters: full tryptic specificity, up to two missed cleavage sites, carbamidomethylation of cysteine residues as fixed modification, and methionine oxidation and protein N-terminal acetylation as variable modifications. Searches were performed using a 20 ppm of precursor ion tolerance and a 20 ppm for fragment ion tolerance. The final reports were then generated after filtering at 1% FDR at both protein and peptide level. Label-free quantification (LFQ) was performed for mouse uterus sample (*e.g.*, the section of “**Application to study cell-type-specific proteomes in mouse uterine tissue**”) with the ‘LFQ-MBR’ workflow provided by Fragpipe, which allowed MSBooster rescoring, MS1-level quantification with IonQuant, match-between-run (MBR), and the MaxLFQ algorithm. For MaxLFQ, a minimum number of ions required for quantifying a protein was set to 2. For MBR, 10 ppm of *m*/*z* tolerance, 0.7 minutes of RT tolerance, and 5% FDR at ion level were applied. The output results were processed and visualized with Perseus and R studio.

Principal component analysis (PCA) was performed using Perseus (v 1.6.15.0). To identify protein signatures in two tissue regions, *t*-test was performed with >1.2-fold difference and *p*-value <0.05. Gene ontology enrichment analysis was performed using a web-based tool, DAVID.^43,44^

## Results and discussion

### The rationale of HD-based sample preparation

Widely-used laser microdissection systems (*e.g.*, Leica LMD or Zeiss PALM Microbeam) use tissue sections that are mounted on PEN membrane-coated slides. Although the PEN membrane greatly facilitates the tissue microdissection process, it creates challenges for efficient protein extraction and digestion in LCM-nanoPOTS workflow. As shown in Fig. 1A, after laser dissection, one side of the tissue pixel is covered by a PEN membrane. After the tissue is catapulted into a DMSO droplet, the tissue side faces to the nanowell surface. In a typical sitting droplet (SD) arrangement, the tissue pixel settles down onto the nanowell surface with the covered PEN membrane (Fig. 1B). The PEN membrane reduces the direct contact of tissue pixel with extraction and digestion buffer, thus reducing the sample recovery for proteomics analysis. To address this problem, we inverted the nanoPOTS chip and performed sample incubation steps with a hanging drop (HD) arrangement (Fig. 1B). The HD method allows the tissue pixel to float in the bottom of the droplet and maximizes the accessibility of extraction and digestion buffer to tissue samples. To directly visualize the difference of HD and SD methods for protein extraction, we captured the images of hematoxylin-stained uterine pixels before and after protein extraction step (Fig. 1C and S1†). As expected, after protein extraction, the color of tissue pixels was lighter for the HD method, while no obvious change was observed for the SD method. Hematoxylin stains nucleic acids in cell nuclei, along with other nucleic acid-containing structures such as rough endoplasmic reticulum or ribosome.^45^ Thus, the decreased hematoxylin color indicated that intracellular structures were efficiently disrupted with HD method.

### Proteome analysis of LCM samples processed by SD and HD methods

**Fig. 2:**
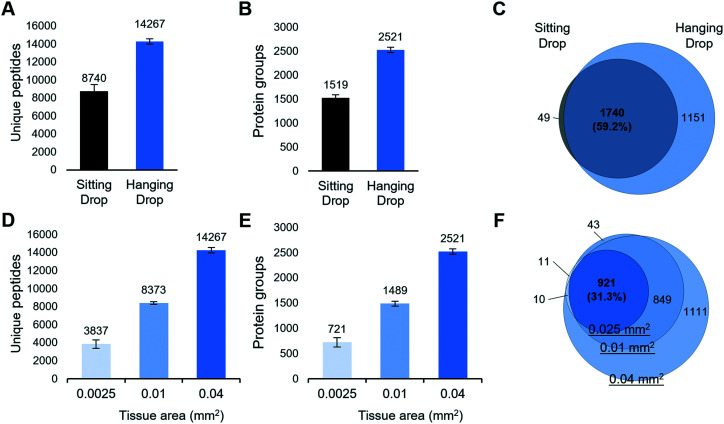
(A–C) Comparison of proteome coverages of hanging droplet and sitting droplet approaches. Numbers of (A) unique peptides and (B) protein groups identified from 0.2 mm × 0.2 mm fresh-frozen mouse liver tissues with a thickness of 10 μm. The error bars indicate standard deviations. (C) Venn diagram showing the overlap of total protein identifications. (D–F) The sensitivity of hanging drop-based spatial proteomics method. Numbers of (D) unique peptides and (E) protein groups identified from 0.05 mm × 0.05 mm, 0.1 mm × 0.1 mm, and 0.2 mm × 0.2 mm mouse liver tissue pixels with a thickness of 10 μm, respectively. (F) Venn diagram of total protein identifications from the three sizes.

To investigate if the HD-based nanoPOTS sample preparation method can improve the performance of spatial proteomics, we dissected 10 μm-thick mouse liver tissues with a size of 0.2 mm × 0.2 mm and analyzed with both SD and HD methods (Table S1†). As shown in Fig. 2A, the average numbers of peptide identifications (*n* = 3) increased from 8750 to 14 267, for the SD and HD methods, respectively, a 63.2% increase. Accordingly, the average numbers of protein identifications were increased from 1519 to 2551, a 66% increase (Fig. 2B). The Venn diagram showed ∼98% of proteins detected by the SD methods were covered by HD method (Fig. 2C). To estimate the overall improvement in protein recovery, we compared the distributions of MS1 peak areas (Fig. S2†). Strikingly, the median of MS1 peak areas for the HD method was ∼7-fold higher than that with the SD method, indicating more protein masses were extracted and digested in HD method. We also evaluated whether the two methods yielded proteins from different cellular localizations. We applied the gene ontology cellular component (GOCC) analysis for the total 1788 and 2890 proteins from SD and HD methods, respectively. As shown in Fig. S3,† the distributions of cellular localization were consistent with both methods.

### Sensitivity evaluation for the HD-based spatial proteomics method

We next evaluated the sensitivity of the LCM-nanoPOTS proteomics platform with the HD method. 10 μm-thick pixels from mouse liver tissue with areas of 0.0025 mm^2^ (0.05 mm × 0.05 mm), 0.01 mm^2^ (0.1 mm × 0.1 mm), and 0.04 mm^2^ (0.2 mm × 0.2 mm) were collected and analyzed (Table S1†). The corresponding cell numbers were approximately 11, 44, and 178 cells, respectively, as calculated from a 10 μm thick section and an average mammalian cell volume of 2250 μm^3^ (Bio Number ID 100434). As shown in Fig. 2D and E, 721 ± 88 (*n* = 4), 1489 ± 48 (*n* = 4), and 2521 ± 52 (*n* = 3) proteins were identified for the smallest to largest tissue pixels, respectively. As expected, proteins identified from smaller tissue pixels were included in larger ones (Fig. 2F). Compared with other spatially resolved proteomic studies of LCM-derived tissues,^23,46^ in which at least 0.5 mm^2^ sized tissues were required to quantify >1000 proteins, the LCM-HD-nanoPOTS system provided >1000 quantifiable proteins from 0.01 mm^2^ sized tissues, *i.e.* using a ∼50-fold smaller tissue volume.

### Optimal surfactant for spatial proteomics

Proper use of a surfactant in cell lysis buffer is not only critical to increase protein solubility from tissue but also to decrease otherwise substantial surface adsorption losses from low-input tissue samples.^47,48^ To identify an optimal surfactant for spatial proteomics, we evaluated three commercially available and MS-compatible surfactants, including DDM, ProteaseMax, and RapiGest (Table S2†). RapiGest and ProteaseMax are widely used in low-input bottom-up proteomics studies,^46,49^ as all have been demonstrated to improve protein solubility and tryptic digestion efficiency. They are also readily degraded under acidic condition, and thus are compatible with a single-tube preparation workflow. DDM is a nonionic and mild surfactant. Our previous studies showed it is compatible with single-tube proteomics workflow, because its elution profile is well separated from peptide peaks. We have used DDM in various nanoPOTS-prepared samples, including mammalian^50^ and plant tissues,^29^ single cultured cells, and primary cells from chick and human donors.^51^ Our studies demonstrated that DDM can efficiently reduce surface adsorption-related sample losses and improve the sensitivity of proteomics analysis. However, the direct comparison of DDM with other MS-compatible surfactants for spatial proteomics has not been made yet.

**Fig. 3:**
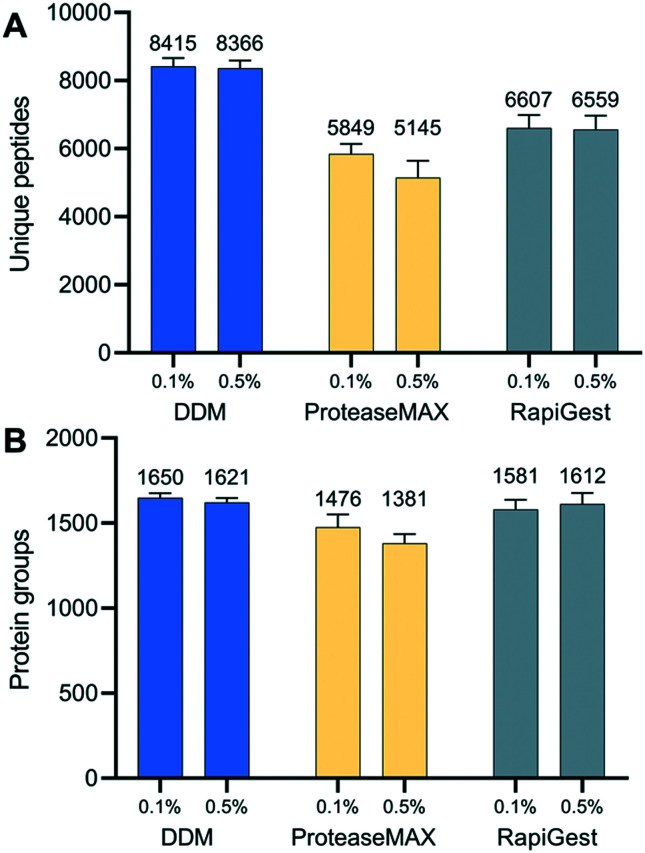
Evaluation of three MS-compatible surfactants for spatial proteomics. (A) Unique peptides and (B) protein groups identified from *n*-dodecyl β-d-maltoside (DDM), ProteaseMAX, and RapiGest SF.

We compared the performance of the three surfactants at two different concentrations (0.1% and 0.5%, m/v) by evaluating proteome coverages and peptide characteristics. Similar to our previous bottom-up proteomics^50^ and top-down proteomics studies,^52^ DDM-based extraction buffers provided the greatest protein coverages, followed by RapiGest and ProteaseMax (Fig. 3). The surfactant concentrations had no significant impact on the peptide and protein identifications, and a lower concentration of 0.1% was sufficient to perform protein extraction. Furthermore, we compared the physicochemical characteristics of identified peptides in each experimental condition. The distributions of GRAVY scores and peptide lengths were slightly higher with the DDM extraction buffer (Fig. S4†), indicating DDM helped to recover more hydrophobic peptides by dynamically coating the nanowell surfaces. We also confirmed no significant bias in cellular protein localization among the three surfactants (Fig. S5†). Based on these results, we chose 0.1% DDM as the optimal extraction buffer for subsequent experiments.

### Application to study cell-type-specific proteomes in mouse uterine tissue

**Fig. 4:**
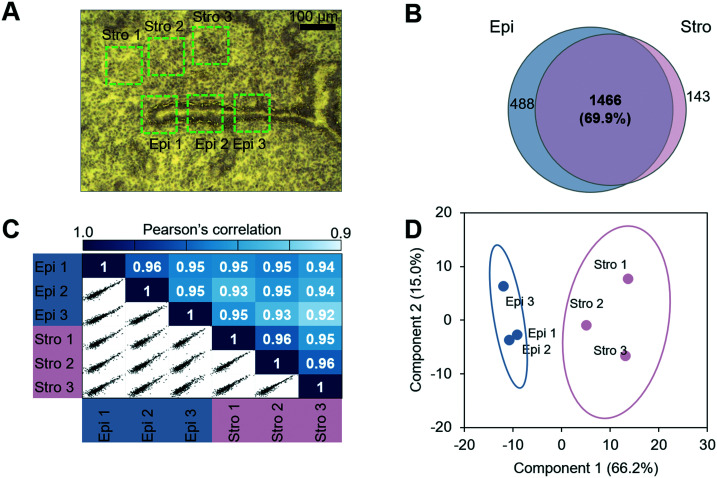
Spatial proteomics analysis of luminal epithelial cells (Epi) and stromal cells (Stro) from mouse uterine tissue sections. (A) An image of hematoxylin-stained tissue section. Total six tissue regions with a lateral dimension of 100 μm × 100 μm and a thickness of 12 μm were dissected and analyzed. (B) The protein identifications, (C) pairwise correlation plots with log 2-transformed LFQ intensities, and (D) PCA projection of the six samples.

We next evaluated the performance of the LCM-HD-nanoPOTS platform for spatial proteomics. We performed a quantitative proteomic measurement of two cell types from mouse uterine tissue sections, including luminal epithelial cells and stromal cells.^27^ We cut the 0.01 mm^2^-sized pixels from two distinct mouse uterus regions (luminal epithelium (Epi)-dominant and stroma (Stro)-dominant) from a 12 μm-thick tissue section (Fig. 4A) (Table S3†). A total of 2097 proteins were identified, and 1466 (69.9%) were commonly quantified in both regions (Fig. 4B and S6†). As expected, higher Pearson correlation coefficients from 0.95 to 0.96 were observed between replicates from the same type of uterus regions, whereas lower correlation coefficients from 0.92 to 0.95 were displayed between different tissue regions (Fig. 4C). To validate protein LFQ data can be used to classify tissue types, we performed principal component analysis (PCA) using commonly quantified proteins (*n* = 603, no missing values) in all the six samples. As shown in Fig. 4D, samples from two tissue types were clearly segregated based on their protein expression with 66.2% of component 1 variance. All three replicates were well clustered together within their corresponding cell types without overlap with the other ellipse. This result suggests that the LCM-nanoPOTS platform with HD method can effectively generate quantitative proteome data to distinguish cell or tissue types.

**Fig. 5:**
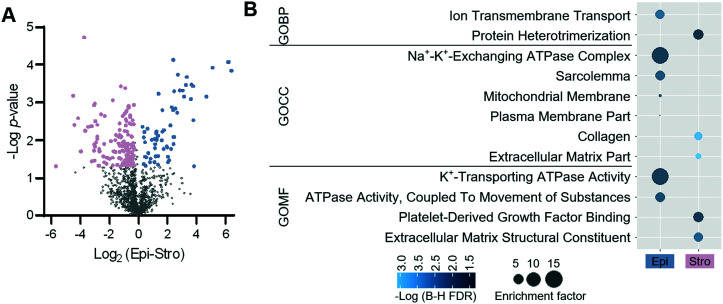
Differentially expressed proteins from the epithelial and stromal dominant regions. (A) Volcano plots of proteins differentially expressed between the two tissue regions. (B) Gene ontology categories obtained from differentially expressed proteins.

To identify protein features specifying the two different tissue types, we performed a *t*-test. Using a fold-change cut-off of 1.2 and a *p*-value cut-off of 0.05, 199 proteins were defined to have significant differences in abundance between the two tissue regions (Fig. 5A). Among the 199 proteins, 62 and 137 proteins were enriched in the luminal epithelium and stroma, respectively. Protein panels enriched from each tissue region were submitted for gene ontology (GO) analysis. Fig. 5B shows the differentially enriched GO categories. As observed in our previous study,^27^ molecular transport GO terms were enriched in the luminal epithelium (ion transmembrane transporter, ion channels, and plasma membrane), while extracellular matrix GO terms were enriched in the stroma (extracellular matrix, collagen, and protein heterodimerization). We also confirmed the enrichment of known protein markers of each region, which were consistent with our previous study (Fig. S7†).^27^ ARVC, RTN4, CD166, VDAC2, ANXA1, K1C19, CTNB1, and ERLN2 showed higher expression in luminal epithelium, while SPA3K, PZP, APOA1, CO1A1, CO6A4, PGBM, EMIL1, PGS2, ALBU, CO3, and IGHM were showing higher expression in the stroma. Together, we demonstrated that the quantitative proteome data achieved *via* LCM-HD-nanoPOTS platform were highly robust to quantify significant protein features in spatial proteomics at the 100 μm spatial resolution.

## Conclusions

Spatial proteomics provides an attractive tool for dissecting complex spatial organizations of cells and microstructures in human tissues related to disease or biological functions. In this work, we introduced the HD-based sample preparation workflow for the LCM-nanoPOTS spatial proteomics platform. We demonstrated the HD arrangement can significantly improve protein extraction and tryptic digestion efficiency, enhancing protein recovery by ∼7 fold. Compared with the commonly-used SD approach, the HD method increased protein identifications by 66% for 0.04 mm^2^ LCM-isolated tissue pixels. We applied the platform to study two different tissue regions from mouse uterus and demonstrated spatial proteomics can be used to identify region or cell-type-specific protein markers. We believe that this platform can be widely used to study functional tissue units and tissue microenvironment-related pathology, and eventually improve disease diagnosis and progression prediction.

Compared with our recent single-cell proteomics studies,^53^ we noted the proteome coverage of the present platform is relatively low, which could be largely attributed to the use of a less powerful Q-Exactive Orbitrap mass spectrometer. Based on our experience, we expect the proteome coverage of the LCM-HD-nanoPOTS-based spatial proteomics would be considerably improved with an advanced MS platform, such as Eclipse Tribrid Orbitrap MS^28^ or TIMS-TOF SCP.^54^ In addition to proteome coverage, the throughput of spatial proteomics can be further improved by integrating isobaric labelling (*e.g.*, Tandem Mass Tag or TMT) to multiplex proteomics analysis, which can be readily integrated into nanoPOTS workflow.^53,55,56^ Together with these improvements, we anticipate the LCM-nanoPOTS-based spatial proteomics will provide a basis for the large-scale mapping of thousands of proteins at single-cell resolution.

## Conflicts of interest

There are no conflicts to declare.

## Supplementary Material

LC-022-D2LC00384H-s001

LC-022-D2LC00384H-s002

## Data Availability

The raw files can be accessed on the ProteomeXchange Consortium *via* the MassIVE partner repository with the data set identifier MSV000089295 and are available at ftp://MSV000089295@massive.ucsd.edu.
