# Supplementary material for: Hanging drop sample preparation improves sensitivity of spatial proteomics
Source: Lab Chip. 2022 Jul 12;22(15):2869–77. doi: 10.1039/d2lc00384h (PMC9320080; doi:10.1039/d2lc00384h)
Supplement: LC-022-D2LC00384H-s001 [file LC-022-D2LC00384H-s001.pdf]

Electronic Supplementary Material (ESI) for Lab on a Chip.
This journal is © The Royal Society of Chemistry 2022

Supplementary Information

Hanging drop sample preparation improves sensitivity of spatial proteomics

Yumi Kwon,[a] Paul D. Piehowski,[a] Rui Zhao,[a] Ryan L. Sontag,[b] Ronald J. Moore,[b] Kristin E. Burnum-Johnson,[a] Richard D. Smith,[b] Wei-Jun Qian,[b] Ryan T. Kelly,[a,c] and Ying Zhu*,[a]

[a]Environmental Molecular Sciences Laboratory, Pacific Northwest National Laboratory, 902 Battelle Boulevard, Richland, WA 99354, USA.

[b]Biological Sciences Division, Pacific Northwest National Laboratory, Richland, WA 99354, USA

[c]Department of Chemistry and Biochemistry, Brigham Young University, C100 BNSN, Provo, UT 84602, USA

* Correspondence: ying.zhu@pnnl.gov

Supplementary Figures

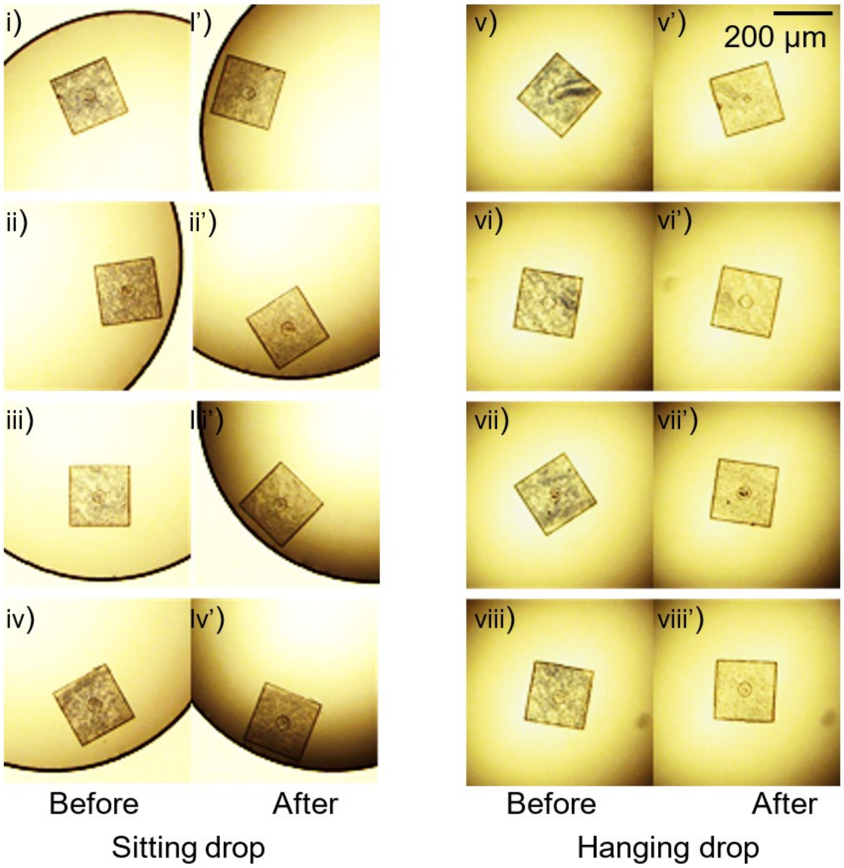

Fig. S1. Microscopic images of LCM-dissected tissue pixels before and after protein extraction step based on sitting drop and hanging drop methods, respectively.

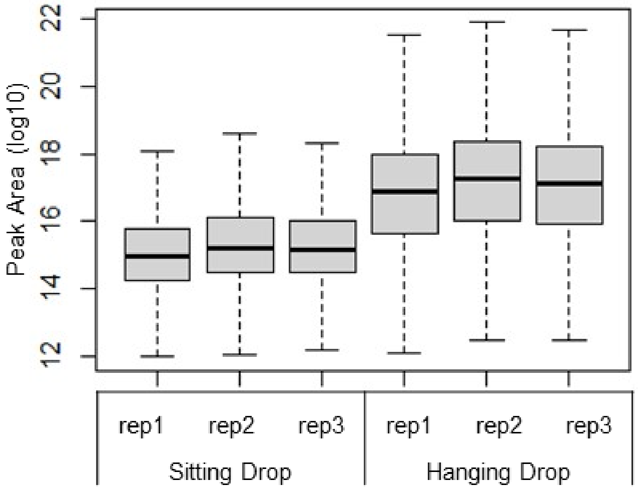

Fig. S2. Comparison of the distributions of peptide peak intensity identified using either SD method or HD method.

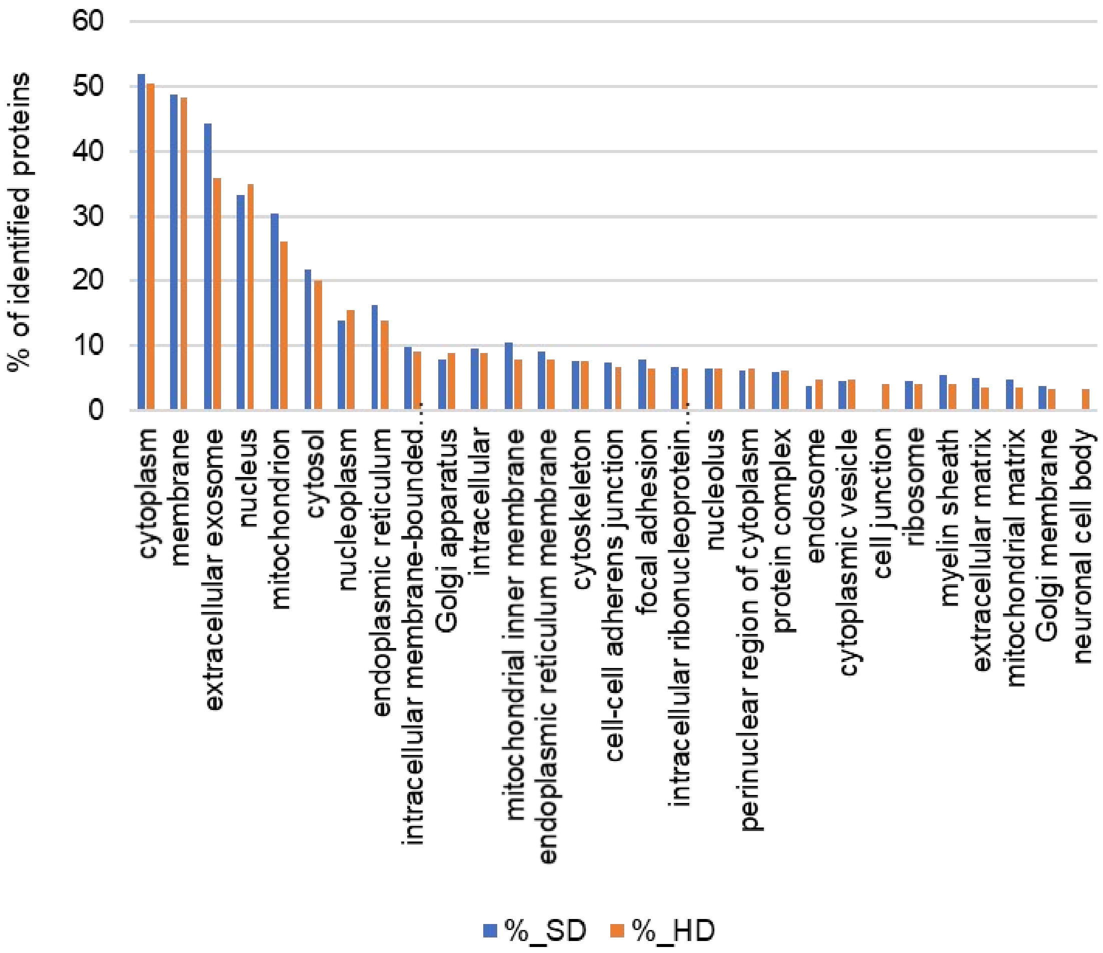

Fig. S3. Gene Ontology Cellular Component (GOCC) distributions of the proteins identified from 0.04 mm^2 mouse liver tissues using SD and HD methods, respectively.

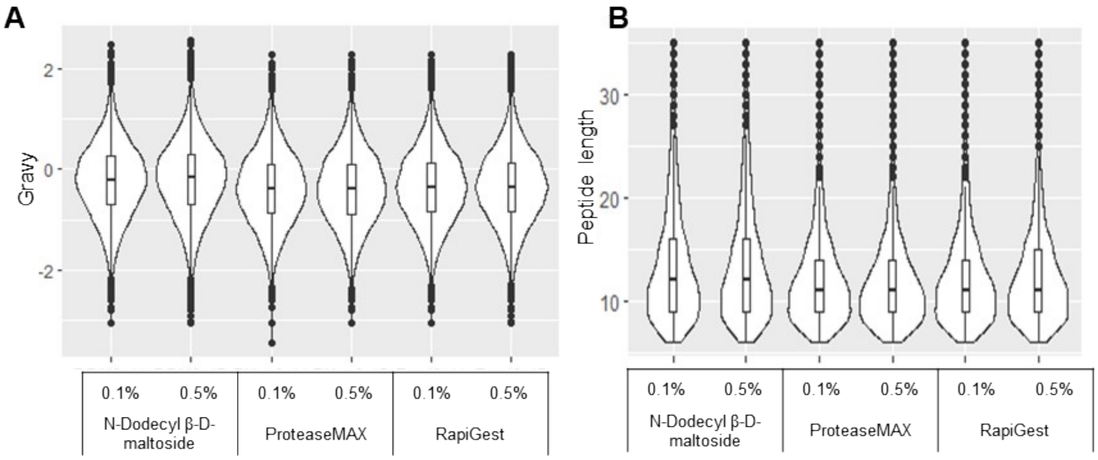

Fig. S4. Distributions of GRAVY scores (A) and peptide lengths (B) for identified peptides using three different surfactants, including DDM, ProteaseMAX, and RapiGest, respectively.

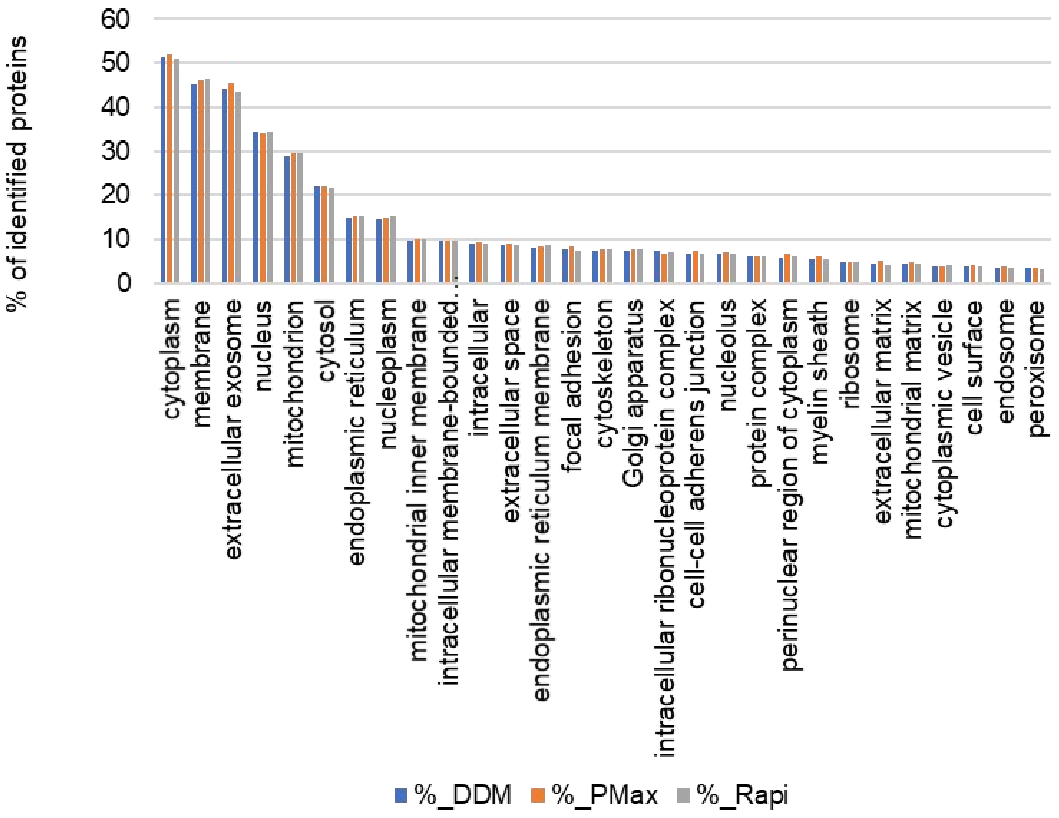

Fig. S5. Gene Ontology Cellular Component (GOCC) distributions of the identified proteins using three different detergents, including DDM, ProteaseMAX, and RapiGest, respectively.

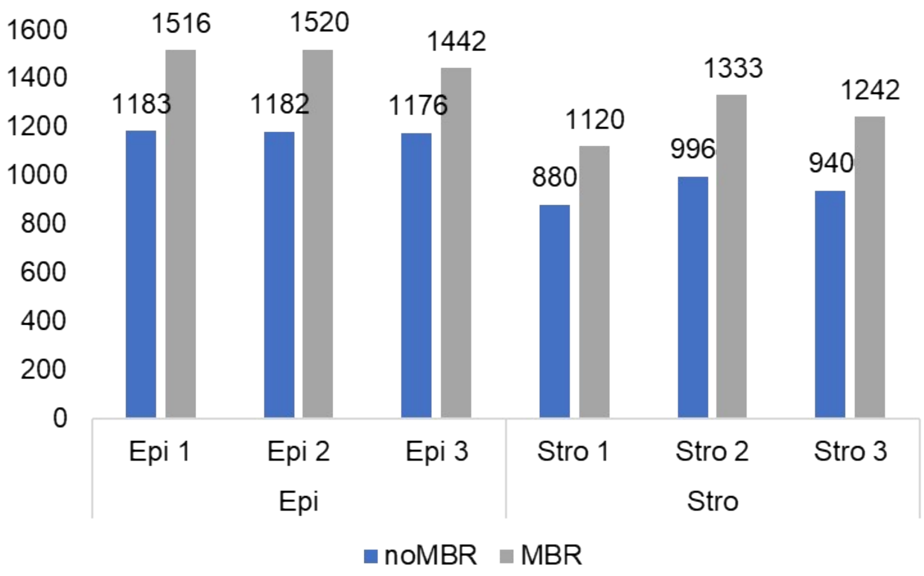

Fig. S6. The numbers of identified proteins for luminal epithelial cells (Epi) and stromal cells (Stro) from mouse uterine tissue sections without and with MBR.

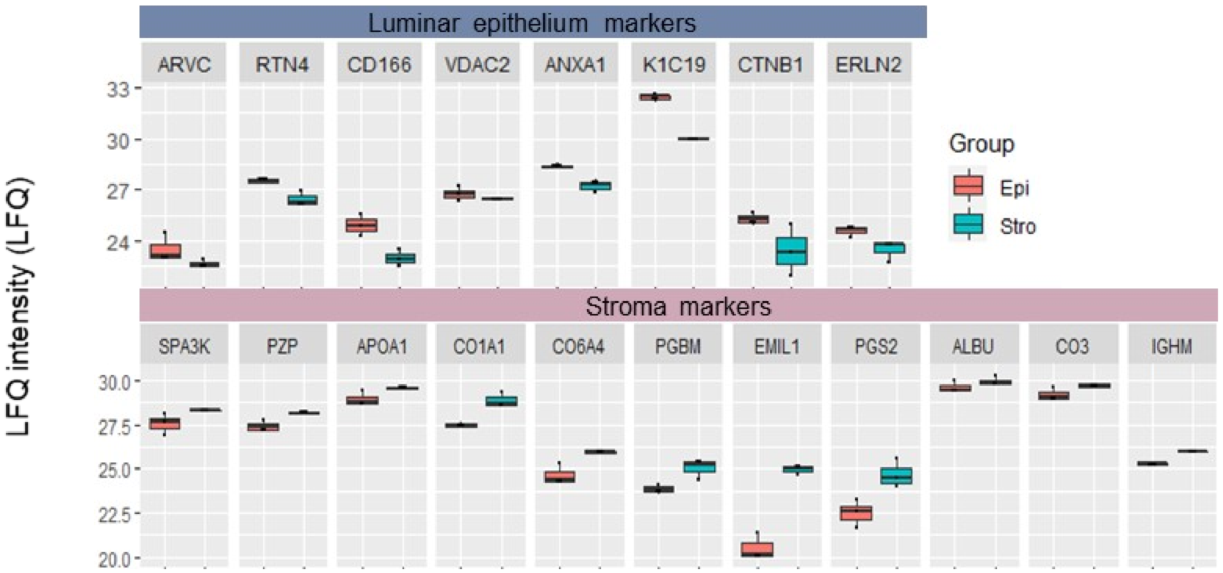

Fig. S7. The abundance differences for proteins enriched in two different tissue regions (luminal epithelium-dominant and stroma-dominant).

Supporting Tables

Table S1. List of proteins identified from mouse liver tissue with hanging drop or sitting drop, and with sizes of 0.0025 mm^2, 0.01 mm^2, and 0.04 mm^2. (Fig. 2)

Table S2. List of proteins identified in the experiment for surfactant comparisons. (Fig. 3)

Table S3. List of proteins identified from mouse uterus tissue samples. (Fig. 4-5)
